# Laparoscopic proximal gastrectomy with esophagogastrostomy using overlap method combined with gastric remnant "U"-shaped fold: a retrospective cohort study

**DOI:** 10.3389/fsurg.2026.1808133

**Published:** 2026-05-29

**Authors:** Yu Zheng, Jia Wang, Chen Wang, Xiangyu Zhang, Shaofan Qiu, Weibo Li, Li Xing, Huadong Wu, Baojun Zhou, Shaowei Ma

**Affiliations:** Department of Gastrointestinal, Hernia and Abdominal Wall Surgery, The Second Hospital of Hebei Medical University, Shijiazhuang, Hebei, China

**Keywords:** anti-reflux surgery, esophagogastrostomy, fundoplication, gastric cancer, laparoscopic surgery, proximal gastrectomy

## Abstract

**Objective:**

Proximal gastrectomy (PG) is increasingly preferred over total gastrectomy for upper gastric cancer to preserve gastric function, yet postoperative reflux remains a critical challenge. The purpose of this study was to compare and analyze the clinical effects and quality of life following totally laparoscopic proximal gastrectomy with esophagogastrostomy using the overlap method combined with a gastric remnant “U” shaped fold (EGOUF) vs. conventional laparoscopic-assisted esophagogastrostomy.

**Methods:**

A retrospective cohort study was conducted involving 60 patients who underwent laparoscopic proximal gastrectomy between January 2023 and March 2025. According to the digestive tract reconstruction method, patients were divided into two groups: the EGOUF group and the non-EGOUF group, each comprising 30 cases. We evaluated the efficacy of the reconstruction method by observing perioperative outcomes, postoperative complications, and short-term nutritional status over a follow-up period of 3–6 months.

**Results:**

After 3 months, the incidence of reflux esophagitis in the EGOUF group was significantly lower than in the non-EGOUF group (6.7% vs. 86.7%, *P* < 0.001). The quality of life in the EGOUF group was superior, with significantly reduced acid reflux and heartburn symptoms (0% vs. 70.0%, *P* < 0.001). After 6 months, the EGOUF group demonstrated better nutritional preservation, including a higher total protein index [66.8 (64.7, 68.5) g/L vs. 62.2 ± 3.6 g/L, *P* < 0.001], higher vitamin B12 levels [566.3 ± 56.0 pg/mL vs. 500.0 (480.0, 532.0) pg/mL, *P* < 0.001], and less weight loss [3.75% ± 6.45% vs. 7.00% (5.00%, 14.00%), *P* = 0.003]. Other short-term nutritional indicators showed no significant differences. Furthermore, the EGOUF group experienced less intraoperative blood loss [15.0 [10.0, 20.0] mL vs. 50.0 [27.5, 50.0] mL, *P* < 0.001] and shorter postoperative hospital stays [9.0 [8.8, 10.3] days vs. 12.0 [10.0, 14.0] days, *P* = 0.001].

**Conclusions:**

The EGOUF technique offers a superior alternative for digestive tract reconstruction following laparoscopic proximal gastrectomy. Compared to the non-EGOUF group, patients undergoing EGOUF experienced significantly lower rates of reflux esophagitis, reduced intraoperative blood loss, quicker postoperative recovery, and better short-term nutritional status, leading to an improved quality of life.

## Introduction

Gastric cancer remains a significant global health burden, with a notable epidemiological shift towards an increased incidence of tumors located in the upper third of the stomach and the esophagogastric junction (EGJ) (1, 2). Traditionally, total gastrectomy (TG) with Roux-en-Y reconstruction has been considered the standard surgical treatment for upper gastric cancer, regardless of the tumor stage. While TG ensures radical tumor removal, it is invariably associated with the complete loss of gastric reservoir function, leading to post-gastrectomy syndromes such as dumping syndrome, severe weight loss, anemia, and nutritional deficiencies, which severely compromise the patients’ postoperative quality of life (1).

In recent years, with the advancements in laparoscopic technology and the increasing emphasis on function-preserving surgery, proximal gastrectomy (PG) has emerged as a viable alternative for early-stage upper gastric cancer (cT1N0M0) and, increasingly, for carefully selected advanced cases (3, 4). Compared to TG, PG preserves the distal half of the stomach, maintaining a portion of the gastric reservoir and the pyloric function, which theoretically contributes to better nutritional status and hormonal regulation (1, 5). However, the major challenge of proximal gastrectomy lies in digestive tract reconstruction. The disruption of the original cardia structure, the Angle of His, and the lower esophageal sphincter (LES), combined with the separation of the vagus nerve during proximal gastrectomy, often results in a high incidence of gastroesophageal reflux disease (GERD) and anastomotic stenosis (6). The loss of the anti-reflux barrier allows gastric acid and bile to flow back into the esophagus, causing severe reflux esophagitis, which can significantly degrade the quality of life despite the preservation of the distal stomach. Furthermore, chronic exposure of the esophageal mucosa to duodenal contents increases the risk of remnant esophageal cancer (7, 8). Therefore, establishing an effective anti-reflux mechanism is the primary objective of reconstruction after proximal gastrectomy (9).

Currently, various anastomotic procedures have been developed to mitigate these complications, including esophagogastrostomy (EG), jejunal interposition (JI), and double-tract reconstruction (DTR) (10–13). While JI and DTR offer reasonable anti-reflux efficacy by diverting bile, they are technically complex, time-consuming, and alter the physiological passage of food, potentially limiting the comprehensive monitoring of the remnant stomach via endoscopy (14, 15). Conversely, direct esophagogastrostomy (EG) is technically simpler and physiologically more natural but has historically been plagued by unacceptably high rates of reflux esophagitis, reported to be as high as 30%–50% in conventional methods (16, 17). To address this, several modified EG techniques incorporating valvuloplasty or fundoplication principles have been introduced, such as the double-flap technique (DFT) (18, 19) and side-overlap esophagogastrostomy (SOFY) (20, 21). Although DFT (Kamikawa procedure) demonstrates excellent anti-reflux performance, it requires intricate hand-sewing skills and a steep learning curve, making it difficult to standardize in laparoscopic settings (22).

There remains a lack of consensus on the optimal reconstruction method that balances technical feasibility with functional outcomes, as different techniques present varying balances between technical complexity and postoperative quality of life (13). For patients with T1-T3 upper gastric cancer, achieving radical resection alongside anti-reflux reconstruction can maximize the retention of the distal gastric volume, reduce the occurrence of short-term postoperative malnutrition, and improve the quality of life by mitigating esophageal reflux. Inspired by the principles of Nissen and Toupet fundoplications used for hiatal hernia repair, we hypothesized that creating a pseudo-fornix around the anastomosis could restore the physiological anti-reflux barrier. For this purpose, we have explored a totally laparoscopic proximal gastrectomy method for digestive tract reconstruction: esophagogastrostomy using the overlap method combined with a gastric remnant “U” shaped fold (EGOUF) (23). This technique aims to prevent esophageal reflux by creating a tunnel-like valve mechanism, accelerate patient recovery, and enhance overall quality of life.

## Materials and methods

### Patients and study design

This study was designed in accordance with the Strengthening the Reporting of Observational Studies in Epidemiology (STROBE) guidelines (24). We retrospectively analyzed the clinical and follow-up data of patients who underwent laparoscopic proximal gastrectomy, comparing the short-term and mid-term clinical effects between the EGOUF group and a control group undergoing conventional laparoscopic esophagogastrostomy (non-EGOUF).

This retrospective cohort study was conducted at the Department of Gastrointestinal, Hernia, and Abdominal Wall Surgery, The Second Hospital of Hebei Medical University. Data were collected from 60 patients who underwent laparoscopic proximal gastrectomy between January 2023 and March 2025. The study protocol was approved by the Institutional Ethics Committee (Approval No.: 2024-R621) and adhered to the ethical principles of the Declaration of Helsinki. Written informed consent was obtained from all patients prior to surgery.

The inclusion criteria were as follows: (1) histologically confirmed adenocarcinoma of the upper third of the stomach or Siewert type II/III adenocarcinoma of the esophagogastric junction (EGJ); (2) clinical stage cT1N0M0 to cT3N1M0 according to the 8th edition of the AJCC/UICC TNM staging system; (3) tumor diameter ≤ 4 cm; (4) no evidence of distant metastasis; and (5) R0 resection achieved. Exclusion criteria included: (1) history of other malignant tumors; (2) preoperative neoadjuvant chemotherapy or radiotherapy; (3) severe cardiopulmonary dysfunction or other underlying diseases prohibiting laparoscopic surgery; (4) conversion to open surgery due to intraoperative complications; and (5) incomplete clinical or follow-up data. Patients were categorized into two groups based on the reconstruction method: 30 patients underwent totally laparoscopic PG with EGOUF (EGOUF group), and 30 patients underwent laparoscopic PG with conventional esophagogastrostomy (non-EGOUF group).

### Surgical procedure

#### Laparoscopic proximal gastrectomy (common steps)

Under general anesthesia, patients were placed in the French position (supine with legs split). The surgeon stood between the patient's legs, with the assistant on the right and the camera operator on the left. A standard five-port method was utilized. Briefly, a 10-mm observation port was placed below the umbilicus, a 12-mm main working port in the left preperitoneal area, a 5-mm auxiliary port in the left anterior axillary line, a 5-mm auxiliary port in the right midclavicular line, and a 5-mm port in the right anterior axillary line for liver retraction. The greater omentum was preserved, while the lymph nodes were dissected strictly according to the Japanese Gastric Cancer Treatment Guidelines (6th edition) for proximal gastrectomy. Specifically, lymph node stations No. 1, 2, 3a, 4sa, 4sb, 7, 8a, 9, 11p, and 11d were dissected (3). The right gastric artery and the gastroepiploic vessels were preserved to maintain blood supply to the remnant stomach. The proximal stomach was fully mobilized, and the distal resection line was determined ensuring a sufficient safety margin.

#### Esophagogastrostomy: EGOUF method (totally laparoscopic)

**Figure 1:**
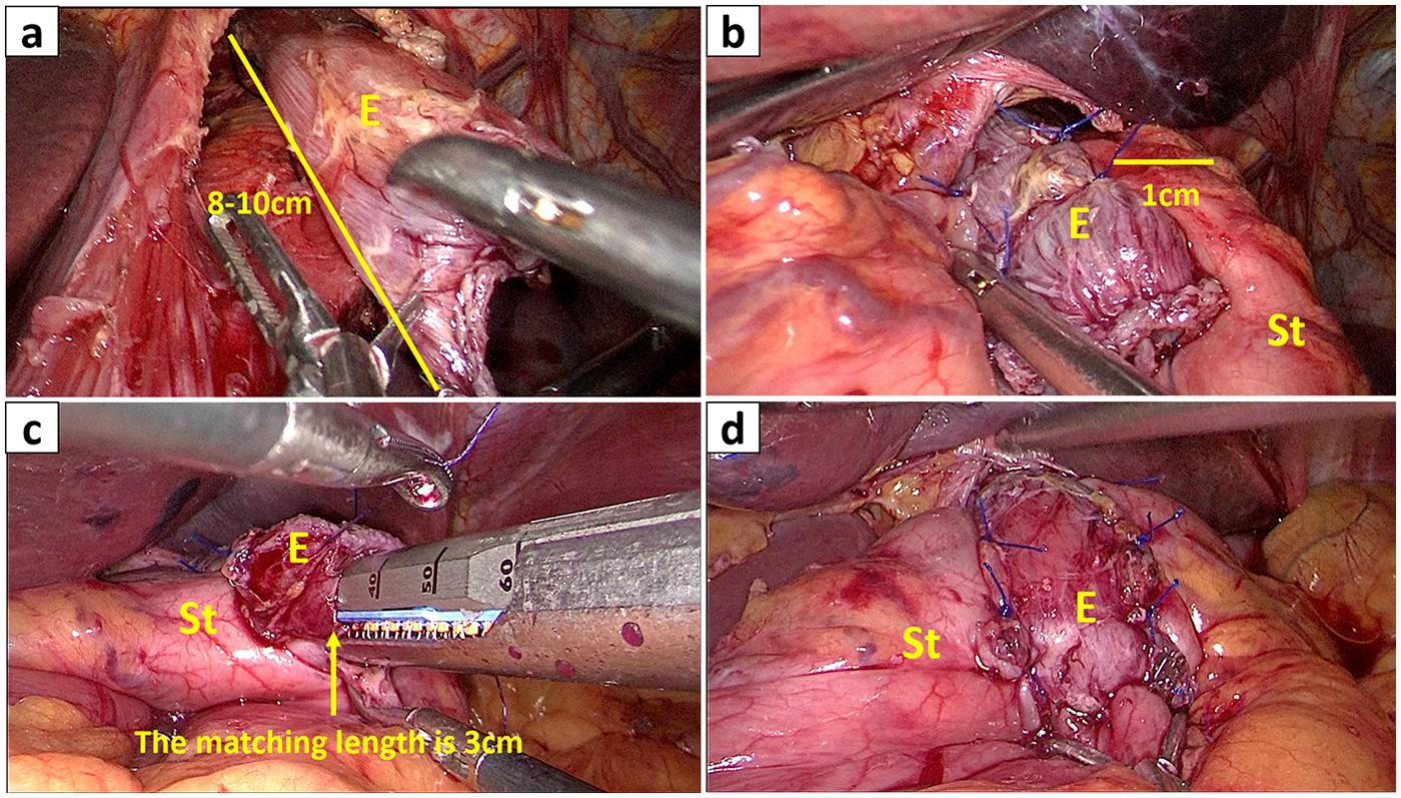
Intraoperative views of the EGOUF procedure. **(a)** Sufficient mobilization of the esophagus (approx. 8-10 cm) into the mediastinum. **(b)** Formation of the "U"-shaped wrap around the lower esophagus. **(c)** Stapled anastomosis using a linear cutter. **(d)** Final view after closure of the entry hole and embedding of the esophageal stump. E: Esophagus; St: Remnant Stomach.

**Figure 2:**
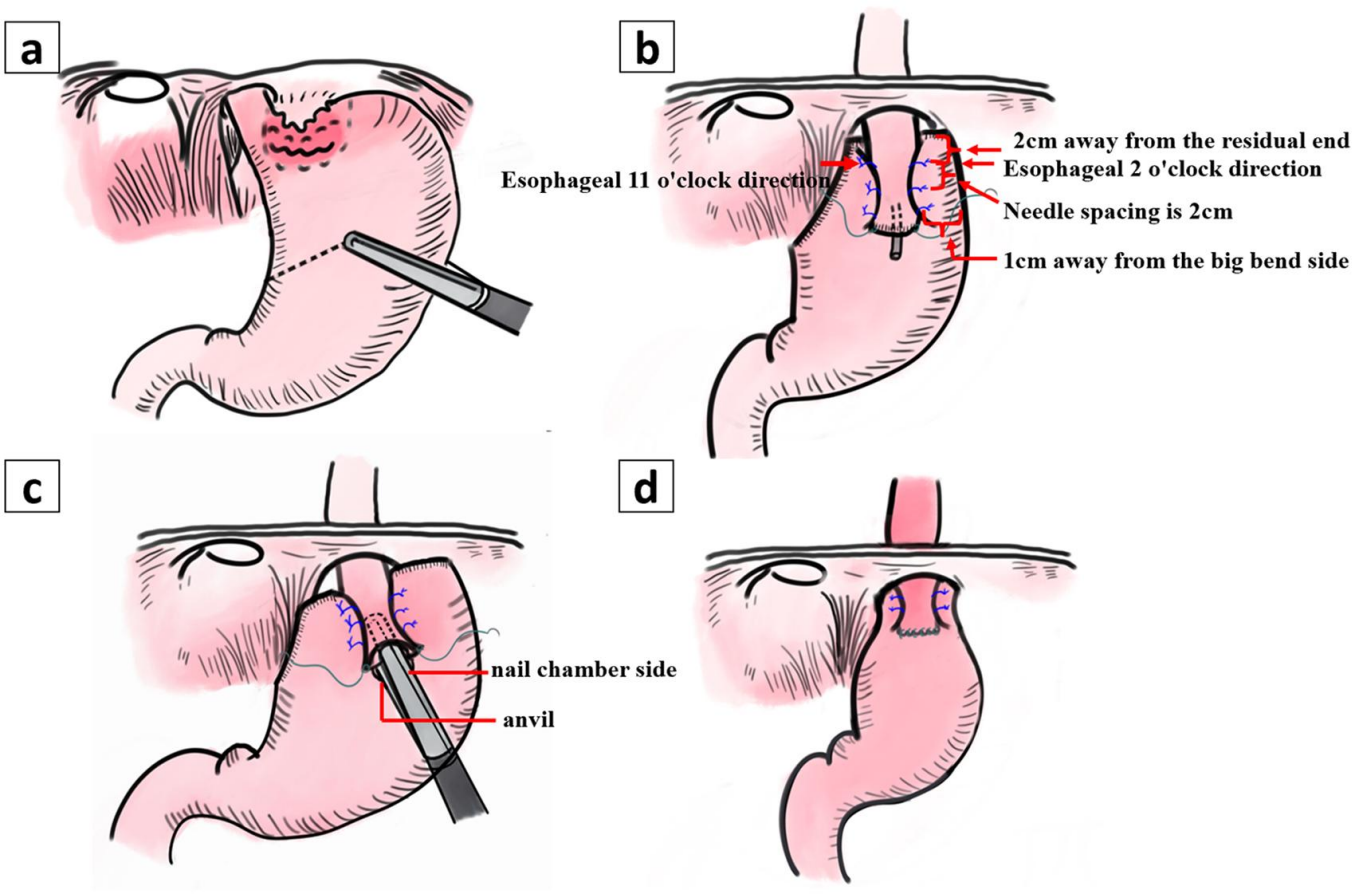
Schematic diagram of the EGOUF procedure. **(a)** The proximal stomach is transected using a laparoscopic linear stapler. The transection line starts from the intersection of the gastroepiploic vessels on the greater curvature and aims 3-5 cm below the lesser curvature of the cardia. **(b)** Construction of the "U"-shaped fold. The anterior wall of the gastric remnant is wrapped around the distal esophagus and fixed with interrupted 2-0 Prolene sutures to create a tunnel-like valve. **(c)** Side-to-side esophagogastrostomy. A linear stapler is used to create the anastomosis between the posterior wall of the esophagus and the anterior wall of the gastric remnant (effective length: approx. 3 cm). The cartridge is inserted into the esophagus, and the anvil into the stomach. **(d)** Completion of the reconstruction. The common entry hole is closed horizontally with 3-0 V-Loc sutures, and the seromuscular layer of the stomach is sutured to the anterior esophageal wall to completely embed the anastomosis.

1. Esophageal Mobilization: The lower esophagus was mobilized approximately 8–10 cm from the cardia into the mediastinum to ensure tension-free anastomosis and sufficient length for the wrapping procedure (Figure 1a).
2. Resection: The proximal stomach was transected using a laparoscopic linear stapler. The transection line was set starting from the intersection of the gastroepiploic vessels on the greater curvature, aiming 3–5 cm below the lesser curvature of the cardia (Figure 2a). The esophagus was transected 2 cm above the EGJ. Intraoperative frozen section analysis was performed when necessary to confirm negative margins.
3. Preparation for Anastomosis: Three “0” V-Loc sutures were pre-placed on both sides of the esophageal stump for traction. A longitudinal enterotomy was made on the posterior wall of the esophageal stump and the anterior wall of the gastric remnant (approximately 4–5 cm from the superior staple line).
4. "U"-Shaped Valvuloplasty: To create the anti-reflux mechanism, the gastric remnant was wrapped around the intra-abdominal esophagus. Using 2–0 Prolene sutures, the left side of the esophagus was sutured to the anterior wall of the gastric remnant at the left anterior aspect relative to the esophagus. Similarly, the right side was fixed at the right anterior aspect. Additional sutures were placed 2 cm and 4 cm distally on both sides to securely wrap the anterior gastric wall around the lower esophagus, forming a "U"-shaped muscular fold (Figures 1b, 2b).
5. Stapled Anastomosis: A laparoscopic linear stapler (45 mm or 60 mm) was inserted. The cartridge limb was introduced into the esophagus, and the anvil limb into the stomach. After positioning, the stapler was fired to create a side-to-side anastomosis (effective length approx. 3 cm) (Figures 1c, 2c).
6. Closure of Common Entry Hole: The common entry hole was closed horizontally using continuous 3–0 V-Loc sutures. Finally, the seromuscular layer of the gastric remnant was sutured to the anterior wall of the esophagus to completely embed the anastomotic site and reinforce the "U"-shaped valve (Figures 1d, 2d). Furthermore, an intraoperative air leak test under saline immersion or a methylene blue test via a gastric tube was routinely performed to confirm the integrity of the anastomosis and ensure there was no leakage. The specimen was extracted through a slightly extended umbilical incision (approx. 4 cm). The detailed surgical procedure is demonstrated in a video (Supplementary file 1).

#### Esophagogastrostomy: non-EGOUF method (conventional laparoscopic reconstruction)

In the control group, patients underwent laparoscopic proximal gastrectomy with conventional digestive tract reconstruction. Following laparoscopic resection and lymphadenectomy as described above, reconstruction was performed using a standard technique without the "U"-shaped fundoplication. The esophagus and gastric remnant were anastomosed typically using a circular stapler (via a mini-laparotomy for anvil insertion and firing) or a linear stapler method (simple side-overlap without the reinforcing fold). Specifically, a 26 mm circular stapler anvil was inserted into the esophageal stump. The stapler body was introduced through a gastrotomy on the anterior wall of the gastric remnant, and the spike was advanced to mate with the anvil. The anastomosis was completed, and the gastrotomy was closed. Critically, no additional fundoplication or wrapping procedures were performed in this group.

### Postoperative management and follow-up

Perioperative outcomes including operative time, reconstruction time, estimated blood loss, and postoperative hospital stay were recorded. Complications were graded according to the Clavien-Dindo classification. Patients were followed up at 3 and 6 months postoperatively. Follow-up assessments included: (1) Upper gastrointestinal radiography and gastroscopy to evaluate anastomotic patency and reflux; (2) The Gastroesophageal Reflux Disease Questionnaire (GerdQ) score; (3) Nutritional parameters (hemoglobin, total protein, albumin, prealbumin, vitamin B12, and BMI). Reflux esophagitis was graded according to the Los Angeles (LA) classification.

### Statistical analysis

Statistical analysis was performed using SPSS version 25.0 (IBM Corp., Armonk, NY, USA). Continuous variables with normal distribution were expressed as mean ± standard deviation (x ± s) and compared using the independent Student's t-test. Non-normally distributed data were expressed as median (interquartile range, IQR: P25, P75) and analyzed using the Mann–Whitney U test. Categorical variables were presented as frequency (percentage) and compared using the Chi-square test or Fisher's exact test. A two-tailed *P*-value < 0.05 was considered statistically significant.

## Results

### Perioperative outcomes and pathological characteristics

There were no statistically significant differences between the EGOUF and non-EGOUF groups regarding baseline characteristics, including gender distribution (*P* = 0.542), age (*P* = 0.065), BMI (*P* = 0.625), and ASA classification (*P* = 0.754) (Table 1). Regarding perioperative outcomes, the EGOUF group demonstrated significantly superior results in several key metrics. The median intraoperative blood loss was significantly lower in the EGOUF group compared to the non-EGOUF group [15.0 [10.0, 20.0] mL vs. 50.0 [27.5, 50.0] mL, *P* < 0.001]. Additionally, the postoperative hospital stay was significantly shorter in the EGOUF group [9.0 [8.8, 10.3] days vs. 12.0 [10.0, 14.0] days, *P* = 0.001]. There were no significant differences in operative time or digestive reconstruction time between the two groups.

**Table 1:** Baseline information, intraoperative conditions, and postoperative and pathological characteristics of patients in the EGOUF and non-EGOUF groups.

Variable^a^	EGOUF Group (*n* = 30)	Non-EGOUF Group (*n* = 30)	*P*-value
Gender			0.542
Male	24 (80.0)	22 (73.3)	
Female	6 (20.0)	8 (26.7)	
Age (years)	68.0 (58.7, 72.0)	69.5 (65.0, 73.0)	0.065
BMI (kg/m^2^)	24.7 (23.7, 25.6)	24.2 (23.7, 25.4)	0.625
ASA Classification			0.754
II	24 (80.0)	23 (76.7)	
III	6 (20.0)	7 (23.3)	
Operative time (min)	190.7 ± 18.6	180.6 ± 24.2	0.073
Reconstruction time (min)	61.2 ± 7.1	55.5 (50.0, 79.3)	0.449
Intraoperative blood loss (mL)	15.0 (10.0, 20.0)	50.0 (27.5, 50.0)	< 0.001
Postoperative hospital stay (days)	9.0 (8.8, 10.3)	12.0 (10.0, 14.0)	0.001
Clavien-Dindo Classification			0.605
Grade I (Anastomotic stenosis)	1 (3.3)	3 (10.0)	
Grade II (Postoperative bleeding)	0 (0)	1 (3.3)	
Tumor size (cm)	1.8 ± 0.9	3.0 (2.4, 3.6)	< 0.001
Pathological T Stage (pT)			0.394
T1a	5 (16.7)	5 (16.7)	
T1b	10 (33.3)	8 (26.7)	
T2	8 (26.7)	5 (16.7)	
T3	7 (23.3)	12 (40.0)	
Harvested Lymph Nodes	20.5 (18.0, 23.5)	19.0 (16.0, 22.0)	0.097
Pathological N Stage (pN)			0.149
N0	24 (80.0)	18 (60.0)	
N1	4 (13.3)	12 (40.0)	
N2	2 (6.7)	0 (0)	
TNM Stage			0.280
Ia	15 (50.0)	13 (43.3)	
Ib	4 (13.3)	3 (10.0)	
IIa	7 (23.3)	4 (13.3)	
IIb	4 (13.3)	10 (33.3)	
Tumor Location (Siewert)			0.436
Type II	15 (50.0)	18 (60.0)	
Type III	15 (50.0)	12 (40.0)	
Differentiation			0.111
Well	6 (20.0)	3 (10.0)	
Moderate	10 (33.3)	7 (23.3)	
Poor	14 (46.7)	20 (66.7)	

a Data are presented as mean ± SD, median (interquartile range), or number (percentage) as appropriate.

Postoperative pathological analysis revealed a significant difference in tumor size, with the EGOUF group having a smaller mean tumor size compared to the non-EGOUF group [1.8 ± 0.9 cm vs. 3.0 (2.4, 3.6) cm, *P* < 0.001]. Other pathological features, including pT stage, pN stage, TNM stage, and tumor differentiation, were comparable between groups. No perioperative mortality or reoperations occurred in either cohort. Complications included one case of anastomotic stenosis in the EGOUF group (3.3%) and three cases in the non-EGOUF group (10.0%), all of which resolved with endoscopic balloon dilation.

### Short-term follow-up (3 months): anti-reflux efficacy

At 3 months postoperatively, the anti-reflux superiority of the EGOUF technique was evident (Table 2). The incidence of reflux esophagitis was significantly lower in the EGOUF group compared to the non-EGOUF group (6.7% vs. 86.7%, *P* < 0.001). In terms of severity according to the Los Angeles classification, the non-EGOUF group exhibited a substantial burden of disease: 30.0% Grade A, 30.0% Grade B, and 26.7% Grade C. In stark contrast, only 6.7% of patients in the EGOUF group had mild (Grade A) esophagitis, with no moderate or severe cases observed. Clinical symptoms correlated with these findings; patients in the EGOUF group reported no acid reflux or heartburn symptoms (GerdQ score median: 0), whereas 70.0% of the non-EGOUF group experienced these symptoms (GerdQ score median: 2.0), requiring proton pump inhibitors (PPIs) in 30.0% of cases (*P* < 0.001).

**Table 2:** Postoperative clinical outcomes at 3 months follow-up.

Variable^a^	EGOUF Group (*n*=30)	Non-EGOUF Group (*n*=30)	*P*-value
Reflux Esophagitis			<0.001
Yes	2 (6.7)	26 (86.7)	
No	28 (93.3)	4 (13.3)	
Los Angeles Classification			<0.001
None	28 (93.3)	4 (13.3)	
Grade A	2 (6.7)	9 (30.0)	
Grade B	0 (0)	9 (30.0)	
Grade C	0 (0)	8 (26.7)	
Grade D	0 (0)	0 (0)	
GerdQ Score	0 (0, 0)	2.0 (1.0, 3.0)	<0.001
Symptoms			
Acid reflux/Heartburn	0 (0)	21 (70.0)	<0.001
Abdominal pain	2 (6.7)	6 (20.0)	0.225
Nausea and vomiting	1 (3.3)	7 (23.3)	0.058
Proton Pump Inhibitor Use	0 (0)	9 (30.0)	0.004

a Data are presented as median (interquartile range) or number (percentage) as appropriate.

**Figure 3:**
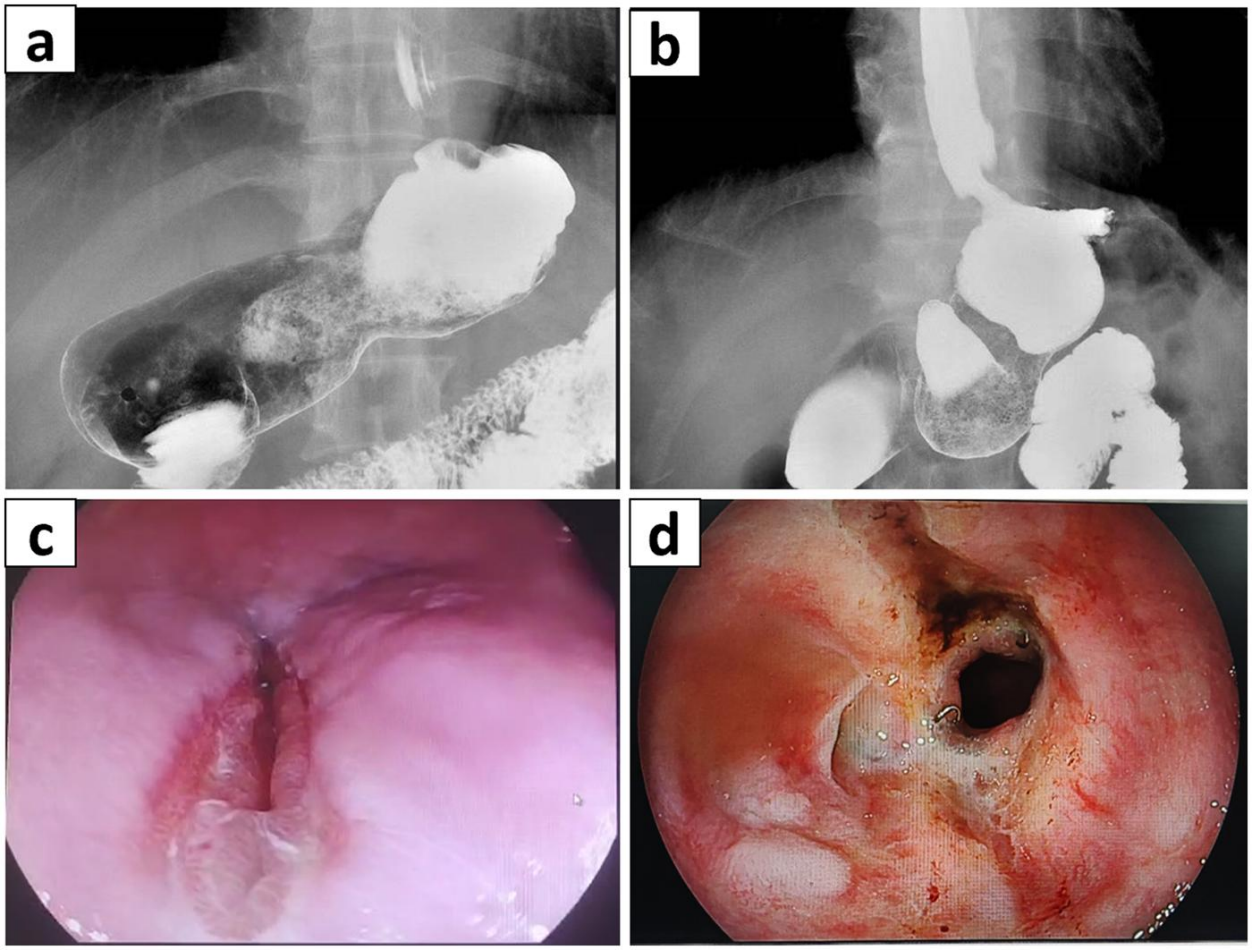
Comparison of postoperative imaging and endoscopic findings at 3 months. **(a)** Upper GI series (supine position) in the EGOUF group showing smooth passage of contrast without reflux. The artificial fundus is visible. **(b)** Upper GI series (supine position) in the non-EGOUF group showing significant reflux of contrast agent from the remnant stomach into the esophagus. **(c)** Gastroscopy in the EGOUF group showing a patent, well-healed anastomosis with no signs of esophagitis. **(d)** Gastroscopy in the non-EGOUF group revealing severe reflux esophagitis with mucosal hyperemia, erosions, and edema at the anastomotic site.

Objective imaging confirmed these clinical observations. Upper gastrointestinal radiography in the EGOUF group demonstrated unobstructed passage of contrast agent with no reflux in the supine position (Figure 3a). Conversely, reflux from the residual stomach into the esophagus was frequently observed in the non-EGOUF group (Figure 3b). Gastroscopy in the EGOUF group revealed a well-healed, patent anastomosis without mucosal injury (Figure 3c), while the control group often showed mucosal hyperemia, erosions, and edema indicative of reflux esophagitis (Figure 3d).

### Short-term follow-up (6 months): nutritional Status

By 6 months post-surgery, the EGOUF group maintained better nutritional status (Table 3). The total protein level was significantly higher in the EGOUF group [66.8 (64.7, 68.5) g/L vs. 62.2 ± 3.6 g/L, *P* < 0.001]. Notably, Vitamin B12 levels were better preserved in the EGOUF group [566.3 ± 56.0 pg/mL vs. 500.0 (480.0, 532.0) pg/mL, *P* < 0.001]. While the absolute BMI values did not differ significantly, the percentage of weight loss was significantly lower in the EGOUF group compared to the control [3.75% ± 6.45% vs. 7.00% (5.00%, 14.00%), *P* = 0.003]. Other indicators such as hemoglobin, albumin, and prealbumin showed no statistically significant differences, although trends favored the EGOUF group. No tumor recurrence was detected in either group during the follow-up period.

**Table 3:** Short-term nutritional status and outcomes at 6 months follow-up.

Variable^a^	EGOUF Group (*n* = 30)	Non-EGOUF Group (*n* = 30)	*P*-value
GerdQ Score	0 (0, 0)	2.0 (1.0, 3.0)	<0.001
Hemoglobin (g/L)	121.9 ± 8.1	117.6 ± 9.3	0.079
Albumin (g/L)	40.2 ± 2.3	38.8 ± 2.9	0.060
Prealbumin (g/L)	0.21 (0.19, 0.22)	0.20 (0.19, 0.21)	0.067
Total Protein (g/L)	66.8 (64.7, 68.5)	62.2 ± 3.6	<0.001
Serum Iron (μmol/L)	13.9 ± 3.9	12.0 (10.0, 13.5)	0.055
Vitamin B12 (pg/mL)	566.3 ± 56.0	500.0 (480.0, 532.0)	<0.001
Total Lymphocyte Count (*10⁹/L)	1.49 (1.30, 1.60)	1.54 (1.42, 1.70)	0.093
BMI (kg/m²)	23.5 (22.9, 24.5)	22.6 ± 2.3	0.057
BMI Percentage Decline (%)	3.75 ± 6.45	7.00 (5.00, 14.00)	0.003
Postoperative Adjuvant Chemotherapy	13 (43.3)	20 (67.7)	0.069
Recurrence	0 (0)	0 (0)	-

a Data are presented as mean ± SD, median (interquartile range), or number (percentage) as appropriate.

## Discussion

The optimal reconstruction strategy following proximal gastrectomy remains a subject of intense debate in upper gastrointestinal surgery. The primary challenge lies in balancing oncological radicality with the functional preservation of the remnant stomach, specifically preventing the debilitating sequelae of reflux esophagitis. In this study, we introduced a modified reconstruction technique, the Esophagogastrostomy using Overlap method combined with gastric remnant “U” shaped fold (EGOUF), and compared its outcomes with conventional laparoscopic reconstruction. Our findings demonstrate that EGOUF significantly reduces the incidence of reflux esophagitis (6.7% vs. 86.7%), minimizes postoperative symptoms, and improves nutritional maintenance compared to standard techniques.

It is noteworthy that the mean tumor size in the EGOUF group was significantly smaller than in the non-EGOUF group. This difference might act as a confounding factor; smaller tumors may necessitate less extensive local dissection, potentially contributing to the observed reduction in intraoperative blood loss and smoother postoperative recovery. However, the anti-reflux benefits and nutritional preservation are mechanistically more likely attributable to the "U"-shaped valve reconstruction rather than tumor size alone.

The exceptionally high rate of reflux in the non-EGOUF group (86.7%) compared to historical cohorts (typically 30%–50%) may be attributed to two factors. First, the conventional linear or circular stapled anastomosis used in our control group lacked any angle of His recreation or anti-reflux wrap, essentially creating a direct, wide-open conduit. Second, the evaluation was performed early at 3 months postoperatively; during this early recovery phase, acute postoperative edema and anatomical remodeling may exacerbate mucosal changes, making the endoscopic presentation appear more severe. The physiological basis for the success of EGOUF lies in its restoration of the anti-reflux barrier. Normal gastroesophageal competence depends on the complex interplay of the lower esophageal sphincter (LES), the crural diaphragm, and the Angle of His (25). Proximal gastrectomy inevitably destroys these structures. Conventional esophagogastrostomy creates a direct conduit between the positive-pressure gastric remnant and the negative-pressure thoracic esophagus, explaining the high reflux rates observed in our control group and historical cohorts (7, 17). The EGOUF technique addresses this by creating a “pseudo-fornix” and a “tunnel-like” valve. The "U"-shaped wrapping of the gastric remnant around the distal esophagus creates a high-pressure zone. When intragastric pressure rises (e.g., during peristalsis or coughing), the wrap compresses the intramural esophagus, preventing retrograde flow. Conversely, the compliance of the wrap allows for bolus passage during swallowing, mimicking the physiological action of the LES.

Comparatively, our technique offers distinct advantages over other emerging anti-reflux procedures. The double-flap technique (DFT), or Kamikawa procedure, is considered the gold standard for anti-reflux efficacy, with reported reflux rates as low as 6%–10% (22, 26). However, DFT is technically demanding, requiring intricate multi-layer hand-suturing that significantly prolongs operative time and has a steep learning curve. In contrast, EGOUF utilizes a linear stapler for the primary anastomosis, simplifying the procedure and reducing the reliance on advanced suturing skills. Our operative times were comparable to standard reconstruction, suggesting that EGOUF is a more reproducible option for general laparoscopic surgeons. Another popular method, the Side Overlap Esophagogastrostomy (SOFY) described by Yamashita et al. (20, 21), also uses a linear stapler but relies primarily on the overlap length for anti-reflux effect. While effective, reflux rates of approximately 17.9% have been reported (21). By adding the "U"-shaped fundoplication to the overlap principle, our EGOUF technique achieved a lower reflux rate (6.7%), suggesting that the physical wrapping provides an additive protective effect beyond simple overlapping.

A critical technical aspect of EGOUF is the extensive mobilization of the esophagus (8–10 cm). Concerns regarding potential ischemia of the esophageal stump due to such extensive dissection are valid. However, the esophageal blood supply is rich and redundant, derived from the inferior thyroid, bronchial, and esophageal arteries, forming a comprehensive intramural and extramural network. In our series, we did not observe any cases of anastomotic leakage or ischemia-related complications. We believe that preserving the adventitial vascular network during mobilization is key. Furthermore, the extensive mobilization allows for a tension-free anastomosis and ensures that the "U"-shaped wrap lies comfortably in the abdomen without being under traction from the diaphragm, which is crucial for the function of the anti-reflux valve.

Nutritionally, the EGOUF group showed superior preservation of total protein and Vitamin B12 levels, and significantly less weight loss. Interestingly, despite identical extents of gastric resection, the EGOUF group demonstrated significantly better nutritional status at 6 months. We hypothesize that this is a direct secondary benefit of reflux prevention. Patients in the non-EGOUF group suffered from severe heartburn and regurgitation, which strongly deters oral intake and promotes a fear of eating. Conversely, the EGOUF group, free from debilitating reflux symptoms, could maintain an adequate and varied diet, facilitating better nutritional recovery. Moreover, preserving the gastric remnant facilitates better iron and Vitamin B12 absorption compared to total gastrectomy, although B12 supplementation may still be necessary due to the loss of parietal cells in the proximal stomach.

Despite these promising results, our study has limitations. First, it is a retrospective, single-center study with a relatively small sample size (*n* = 60), which may introduce selection bias. Second, our endoscopic evaluation for reflux was conducted at 3 months postoperatively. While this captures early postoperative changes, patients are still in the recovery and remodeling phase. Generally, a definitive assessment of chronic reflux esophagitis requires gastroscopy at 1 year post-surgery. Therefore, the long-term anti-reflux durability of EGOUF remains to be validated. Third, we relied on GerdQ scores and endoscopy to assess reflux. Objective physiological testing, such as 24-hour pH impedance monitoring, would provide more definitive evidence of the anti-reflux mechanism's efficacy and should be included in future prospective trials. Furthermore, the evaluation of new surgical innovations requires specialized, phased pathways distinct from pharmacological trials. According to the IDEAL (Idea, Development, Exploration, Assessment, Long-term study) framework (27), our current study represents an early ‘Development/Exploration’ stage (Stage 2a/2b) aimed at assessing safety, refining the technique, and observing short-term efficacy. Finally, the comparison with a non-standardized control group (varying between circular and linear staplers without fundoplication) reflects the real-world evolution of our practice but introduces heterogeneity.

## Conclusion

In conclusion, Laparoscopic Proximal Gastrectomy with EGOUF reconstruction is a safe, feasible, and effective surgical option for upper gastric cancer. By combining a linear stapled anastomosis with a "U"-shaped valvuloplasty, this technique provides an effective anti-reflux barrier that significantly reduces postoperative esophagitis and improves quality of life compared to conventional reconstruction. It offers a balanced solution that preserves gastric function while ensuring short-term oncological outcomes and patient comfort. Future multi-center prospective randomized controlled trials are warranted to validate these findings and establish EGOUF as a standard of care.

## Data Availability

The original contributions presented in the study are included in the article/Supplementary Material, further inquiries can be directed to the corresponding authors.

## Supplementary material

Supplementary File 1 Video demonstration of the EGOUF procedure. This video illustrates the step-by-step technique of totally laparoscopic proximal gastrectomy with EGOUF reconstruction. Key sequences include esophageal mobilization, formation of the "U"-shaped anti-reflux fold, side-to-side linear stapled anastomosis, and seromuscular embedding of the esophageal stump.

## References

[1] Hipp J Hillebrecht HC Kalkum E Klotz R Kuvendjiska J Martini V. Systematic review and meta-analysis comparing proximal gastrectomy with double-tract-reconstruction and total gastrectomy in gastric and gastroesophageal junction cancer patients: still no sufficient evidence for clinical decision-making. Surgery. (2023) 173(4):957–67. 10.1016/j.surg.2022.11.018 36543733

[2] Kumamoto T Kurahashi Y Niwa H Nakanishi Y Okumura K Ozawa R. True esophagogastric junction adenocarcinoma: background of its definition and current surgical trends. Surg Today. (2020) 50(8):809–14. 10.1007/s00595-019-01843-4 31278583

[3] Japanese Gastric Cancer Association. Japanese Gastric cancer treatment guidelines 2021 (6th edition). Gastric Cancer. (2023) 26(1):1–25. 10.1007/s10120-022-01331-8 36342574 PMC9813208

[4] Japanese Gastric Cancer Association. Japanese Gastric cancer treatment guidelines 2018 (5th edition). Gastric Cancer. (2021) 24(1):1–21. 10.1007/s10120-020-01042-y 32060757 PMC7790804

[5] Sun Y Chen C Hou L Zhao E. Short-term outcomes and quality of life of esophagogastrostomy versus the double-tract reconstruction after laparoscopic proximal gastrectomy. BMC cancer. (2024) 24(1):1324. 10.1186/s12885-024-13095-8 39468480 PMC11520072

[6] Shi M Hu Z Wu K Yang D Fu H Zhang J. Comparative study of pyloromyotomy and H-M pyloroplasty in proximal gastrectomy for adenocarcinoma of esophageal-gastric junction. J Gastrointest Surg. (2022) 26(8):1585–95. 10.1007/s11605-022-05347-4 35585422

[7] Katsoulis IE Robotis JF Kouraklis G Yannopoulos PA. What is the difference between proximal and total gastrectomy regarding postoperative bile reflux into the oesophagus? Dig Surg. (2006) 23(5-6):325–30. 10.1159/000097948 17164544

[8] Theisen J Peters JH Fein M Hughes M Hagen JA Demeester SR. The mutagenic potential of duodenoesophageal reflux. Ann Surg. (2005) 241(1):63–8. 10.1097/01.sla.0000150072.55037.e3 15621992 PMC1356847

[9] Gao P He XL Han Z. Evaluation of esophagogastric anastomosis with additional mechanical anti-reflux barrier after proximal gastrectomy. Zhonghua Wei Chang Wai Ke Za Zhi. (2024) 27(10):1018–26. 10.3760/cma.j.cn441530-20240731-00267 39428223

[10] Du N Wu P Wang P Du Y Li K Wang Z. Reconstruction methods and complications of esophagogastrostomy and jejunal interposition in proximal gastrectomy for gastric cancer: a meta-analysis. Gastroenterol Res Pract. (2020) 2020:8179254. 10.1155/2020/8179254 32411203 PMC7201443

[11] Ichikawa D Ueshima Y Shirono K Kan K Shioaki Y Lee CJ. Esophagogastrostomy reconstruction after limited proximal gastrectomy. Hepato-gastroenterology. (2001) 48(42):1797–801. 11813627

[12] Nishimura E Irino T Matsuda S Fukuda K Nakamura R Kawakubo H. Comparison of changes in body-fat mass and reflux esophagitis among reconstruction methods for proximal gastrectomy. Asian J Surg. (2023) 46(1):394–8. 10.1016/j.asjsur.2022.04.110 35570106

[13] Li B Wang Y Li B Shan F Li Z. Short-term outcomes and long-term quality of life of reconstruction methods after proximal gastrectomy: a systematic review and meta-analysis. BMC cancer. (2024) 24(1):56. 10.1186/s12885-024-11827-4 38200411 PMC10777503

[14] Isobe T Hashimoto K Kizaki J Matono S Murakami N Kinugasa T. Reconstruction methods and complications in proximal gastrectomy for gastric cancer, and a comparison with total gastrectomy. Kurume Med J. (2014) 61(1-2):23–9. 10.2739/kurumemedj.MS64003 25152248

[15] Yabusaki H Kodera Y Fukushima N Hiki N Kinami S Yoshida M. Comparison of postoperative quality of life among three different reconstruction methods after proximal gastrectomy: insights from the PGSAS study. World J Surg. (2020) 44(10):3433–40. 10.1007/s00268-020-05629-5 32506229 PMC7458934

[16] [Chinese consensus on digestive tract reconstruction after proximal gastrectomy (2024 edition)]. Zhonghua Wei Chang Wai Ke Za Zhi. (2024) 27(10):983–95. 10.3760/cma.j.cn441530-20240918-00323 39428219

[17] Shaibu Z Chen Z Mzee SAS Theophilus A Danbala IA. Effects of reconstruction techniques after proximal gastrectomy: a systematic review and meta-analysis. World J Surg Oncol. (2020) 18(1):171. 10.1186/s12957-020-01936-2 32677956 PMC7367236

[18] Kuroda S Nishizaki M Kikuchi S Noma K Tanabe S Kagawa S. Double-Flap technique as an antireflux procedure in esophagogastrostomy after proximal gastrectomy. J Am Coll Surg. (2016) 223(2):e7–e13. 10.1016/j.jamcollsurg.2016.04.041 27157920

[19] Kuroda S Ishida M Choda Y Muraoka A Hato S Kagawa T. A multi-center, prospective, clinical study to evaluate the anti-reflux efficacy of laparoscopic double-flap technique (lD-FLAP study). Ann Gastroenterol Surg. (2024) 8(3):374–82. 10.1002/ags3.12783 38707222 PMC11066497

[20] Yamashita Y Yamamoto A Tamamori Y Yoshii M Nishiguchi Y. Side overlap esophagogastrostomy to prevent reflux after proximal gastrectomy. Gastric Cancer. (2017) 20(4):728–35. 10.1007/s10120-016-0674-5 27942874

[21] Yamashita Y Tatsubayashi T Okumura K Miyamoto T Ueno K. Modified side overlap esophagogastrostomy after laparoscopic proximal gastrectomy. Ann Gastroenterol Surg. (2022) 6(4):594–9. 10.1002/ags3.12549 35847432 PMC9271030

[22] Shibasaki S Suda K Nakauchi M Kikuchi K Kadoya S Ishida Y. Robotic valvuloplastic esophagogastrostomy using double flap technique following proximal gastrectomy: technical aspects and short-term outcomes. Surg Endosc. (2017) 31(10):4283–97. 10.1007/s00464-017-5489-x 28364148

[23] Wang L Ma H Ren P Chang H Wang Y Chen Y. A novel esophagogastrostomy technique for laparoscopic proximal gastrectomy: conical remnant gastroesophageal Side-overlap fundoplication. Cancer Diagnosis & Prognosis. (2023) 3(5):609–15. 10.21873/cdp.10263 37671301 PMC10475915

[24] von Elm E Altman DG Egger M. The strengthening the reporting of observational studies in epidemiology (STROBE) statement: guidelines for reporting observational studies. J Clin Epidemiol. (2007) 60(6):344–9. 10.1016/j.jclinepi.2007.11.008 18313558

[25] Fujii Y Yasuda T Inoue T. Laparoscopic esophagogastric anastomosis with stapled Pseudo-Fornix for reflux esophagitis prevention after proximal gastrectomy. Cureus. (2022) 14(6):e25561. 10.7759/cureus.25561 35784962 PMC9247743

[26] Jingtao Z Shaoqin C Tao Z Li Y Sheng Y Qingqi H. Clinical outcomes of double-flap technique versus gastric tube reconstruction following laparoscopic proximal gastrectomy: a multicenter propensity score-matched cohort study. World J Surg Oncol. (2025) 23(1):110. 10.1186/s12957-025-03672-x 40158150 PMC11954176

[27] Li B Wang Y Shan F Li S Jia Y Xue K. Surgical safety and technical optimization of arch-bridge anastomosis after laparoscopy-assisted proximal gastrectomy: an IDEAL stage 2a prospective cohort study. Chin Med J. (2026) 139:1411–3. 10.1097/CM9.0000000000003907 41498181 PMC13120632

